# Advances in the use of nanomaterials for nucleic acid detection in point-of-care testing devices: A review

**DOI:** 10.3389/fbioe.2022.1020444

**Published:** 2022-10-13

**Authors:** Ziyu He, Changsheng Liu, Zhongyu Li, Zhou Chu, Xiang Chen, Xupeng Chen, Yuan Guo

**Affiliations:** ^1^ Department of Scientific Research, Zhuzhou Hospital Affiliated to Xiangya School of Medicine, Central South University, Zhuzhou, China; ^2^ Department of Blood Transfusion, Zhuzhou Hospital Affiliated to Xiangya School of Medicine, Central South University, Zhuzhou, China; ^3^ Department of Clinical Laboratory, Zhuzhou Hospital Affiliated to Xiangya School of Medicine, Central South University, Zhuzhou, China; ^4^ Department of Cardiovascular Medicine, Zhuzhou Hospital Affiliated to Xiangya School of Medicine, Central South University, Zhuzhou, China

**Keywords:** nanomaterial, nucleic acid detection, point-of-care testing devices, sensor, nucleic acid enrichment

## Abstract

The outbreak of the coronavirus (COVID-19) has heightened awareness of the importance of quick and easy testing. The convenience, speed, and timely results from point-of-care testing (POCT) in all vitro diagnostic devices has drawn the strong interest of researchers. However, there are still many challenges in the development of POCT devices, such as the pretreatment of samples, detection sensitivity, specificity, and so on. It is anticipated that the unique properties of nanomaterials, e.g., their magnetic, optical, thermal, and electrically conductive features, will address the deficiencies that currently exist in POCT devices. In this review, we mainly analyze the work processes of POCT devices, especially in nucleic acid detection, and summarize how novel nanomaterials used in various aspects of POCT products can improve performance, with the ultimate aims of offering new ideas for the application of nanomaterials and the overall development of POCT devices.

## Introduction

Point-of-care testing (POCT), which refers to detection immediately after sampling—without the need for professional personnel and equipment—has the advantages of being in real-time, fast, low cost, and so on (Buckle et al., 2021; Xu et al., 2021). Operators do not need to be medical professionals, and testing does not need to be in a fixed site, but can take place anywhere, e.g., in an emergency room, ambulance, outdoors or in the home (Ferreira et al., 2018; Yan et al., 2021). WHO has defined the criteria for POCT products as “ASSURED” (i.e., affordable, sensitive, specific, user-friendly, rapid and robust, equipment-free, and deliverable to end-users) (Land et al., 2019). However, there is a challenge in fulfilling these criteria, especially the sensitivity and specificity of the device. Thus, novel methods are urgently needed to ensure the rapid development of POCT devices.

Nanomaterials can be defined as materials possessing at least one external dimension within the nanometer scale of 1–100 nm (Suan Ng et al., 2022). Due to their special features, nanomaterials offer a wide range of possibilities in various fields, such as biocompatibility, fluorescence, electrical and thermal conductivity, and magnetism (Guo et al., 2018; Li et al., 2020; Tang et al., 2020; Gong N et al., 2021; Li J et al., 2022a). Meanwhile, nanomaterials have different shapes and can be divided into four types: 0D (e.g., spherical nanomaterials), 1D (e.g., nanotubes and nanowires), 2D (e.g., graphenes), and 3D (e.g., nanoprisms and nanoflowers). In line with their advantages and disadvantages, nanomaterials have been applied in different fields, such as the diagnosis of disease, detection of metal ions, and so on (Lai et al., 2018; Quesada-Gonzalez et al., 2018; Ma et al., 2020; Li J et al., 2021). Owing to their specific features, nanomaterials have also become integral in enhancing the performance of POCT devices.

In recent years, nanomaterials have been applied in multiple POCT devices to improve the efficiency of nucleic acid detection, especially in the face of the coronavirus (COVID-19) pandemic. Kim et al. (2021) found nanomaterial-based thin films were superior transducers that can reliably detect about 100 copies of the virus without being disturbed by interfering agents in a virus culture medium (WHO considers a viral load under 10^6^ copies/mL negative). Interestingly, the relations between various thin film properties and the sensitivity of immunosensors were systematically analyzed using a special calculation method that showed good correlation. Hasanzadeh et al. (2021) introduced the application of nanomaterials in protective equipment, the loading and delivery of vaccines, and COVID-19 detection equipment. Xiao C et al. (2022) also systematically elaborated relevant studies regarding the use of nanomaterials in preventing viral spread, preparing vaccines, and diagnosing coronavirus. These works show that nanomaterials have penetrated all stages in the diagnosis and treatment of COVID-19, and hence their integration with POCT devices will be an increasing trend in the future detection market.

A clear understanding of the core technology of POCT devices is of paramount importance. The sensor as receiver controls the accuracy and sensitivity of the devices, because it can register the measured information and output as an electrical signal or in other required forms. A nanosensor is also a type of sensor, and has the advantages of miniaturization, digitalization, intelligence, multi-functionality, systematization, and network capacity, which shows its good application prospects in the development of POCT devices (Yang et al., 2018; Wu et al., 2020b; Li Q et al., 2021; Suan Ng et al., 2022; Xiao M. F et al., 2022). As Mitasha has described, using various biomolecules to detect specific analytes, activatable sensors in POCT products can be divided into immunological-based tests, nucleic acid (NA)-based tests, and other biomarker-based tests (Bharadwaj et al., 2021). Activatable sensors can improve specificity and sensitivity compared to traditional detection, hence providing more comprehensive information and obtaining faster detection speed (Wiraja et al., 2019; Ma et al., 2020; Xie et al., 2020). Therefore, the advantages of activatable sensors all meet the demands for POCT, and are widely used in POCT devices.

The NA-based nanosensor is an activatable sensor, which not only targets NA or proteins (e.g. aptamer), but can also combine antisense oligonucleotides or interfering RNAs to serve both therapeutic and diagnostic purposes (theragnostic) (Liu et al., 2017; Xie et al., 2020; He et al., 2021; Khan et al., 2021). Use of an NA-based sensor as a screening tool to detect COVID-19 produces highly specific and sensitive results (Dronina et al., 2021; Zhang M et al., 2022). In recent decades, the NA-based sensor has been used as a detector to assess heavy metal ion concentration, where it has also demonstrated good performance (Liu et al., 2018; Guo et al., 2021; Chen et al., 2022). In summary, deeper insights into POCT devices and their application include disease diagnosis and therapy, pathogen diagnosis, heavy metal ion monitoring, and so on (Cho et al., 2018; Zeng et al., 2020; Kim H et al., 2021; Mo et al., 2021; Jani and Peter., 2022). However, using a NA-based sensor to achieve the purpose of detection in POCT, to meet the abovementioned WHO-defined criteria, requires greater accuracy and shorter response times, and can, to a certain extent, be achieved by combination with nanomaterials.

**FIGURE 1:**
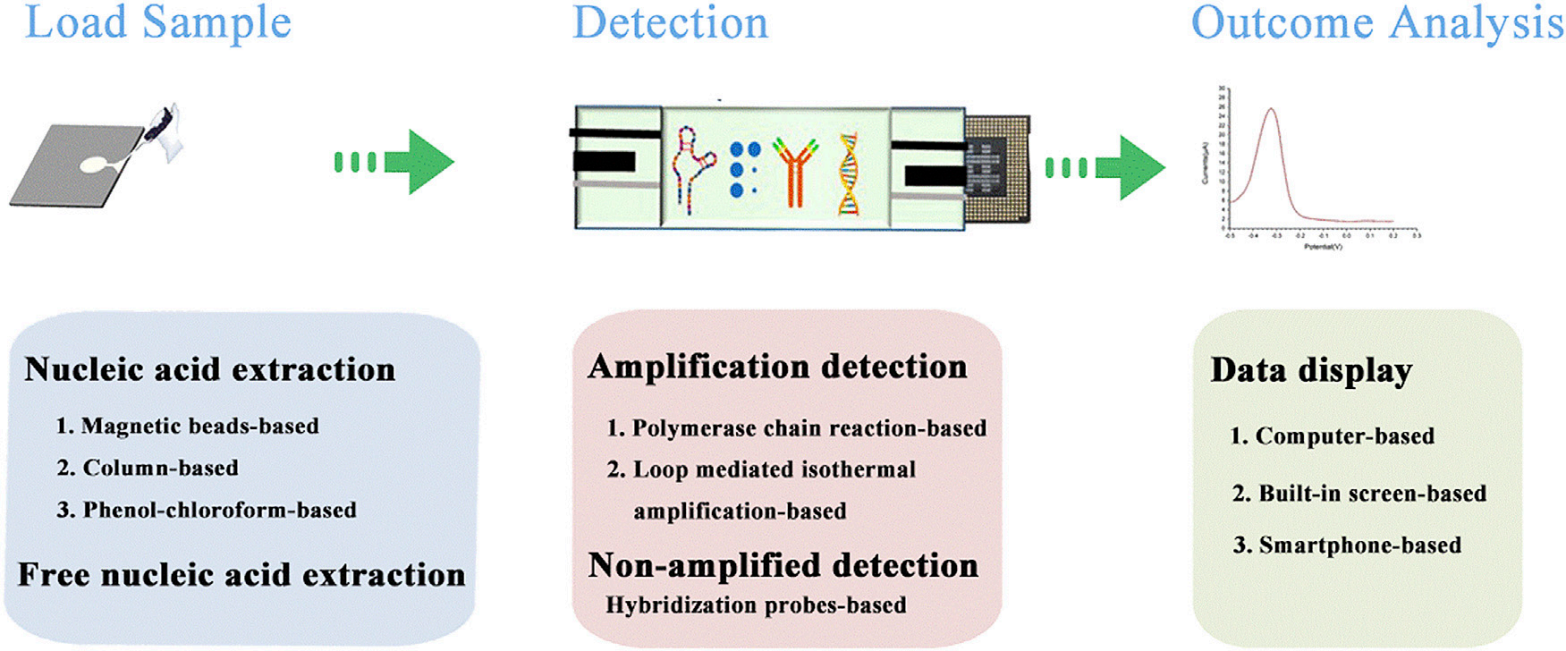
The schematic diagram of POCT device detection process.

In this review, we firstly clarify the current status and trends in POCT in recent years to elicit the latest information, as these aspects have been broadly reviewed in general terms (Prattis et al., 2021; Zhang N et al., 2022). Moreover, we focus on introducing the role of nanomaterials in the work processes of POCT devices, as illustrated in Figure 1, including loading the sample, detection, and data display. In relation to detection especially, we elaborate two functions: amplification and non-amplification, and highlight the important role of nanomaterials in these functions. Overall, this work aims to promote further development of POCT devices, and in particular, POCT devices related to nucleic acid detection. It also offers new ideas for the continued development of POCT devices with high sensitivity, low assay cost, and low power requirements.

## Loading the sample

To reduce interference material in the sample, and to avoid influence on the determination of the result, sample purification is an important step that cannot be ignored. Currently, for the extraction and purification of NA, common methods are magnetic bead-based, column-based, and phenol-chloroform-based (Mou et al., 2019; Wang S et al., 2021; White et al., 2022). In the early days of POCT devices, few researchers considered the project of integrating sample preprocessing into POCT devices. It is well known that the complex and time-consuming sample preparation process should be performed by professionals, because direct loading of samples under unpurified conditions can cause high false positives and negative results. These processes are difficult to promote, which hinders further development of POCT technology.

**FIGURE 2:**
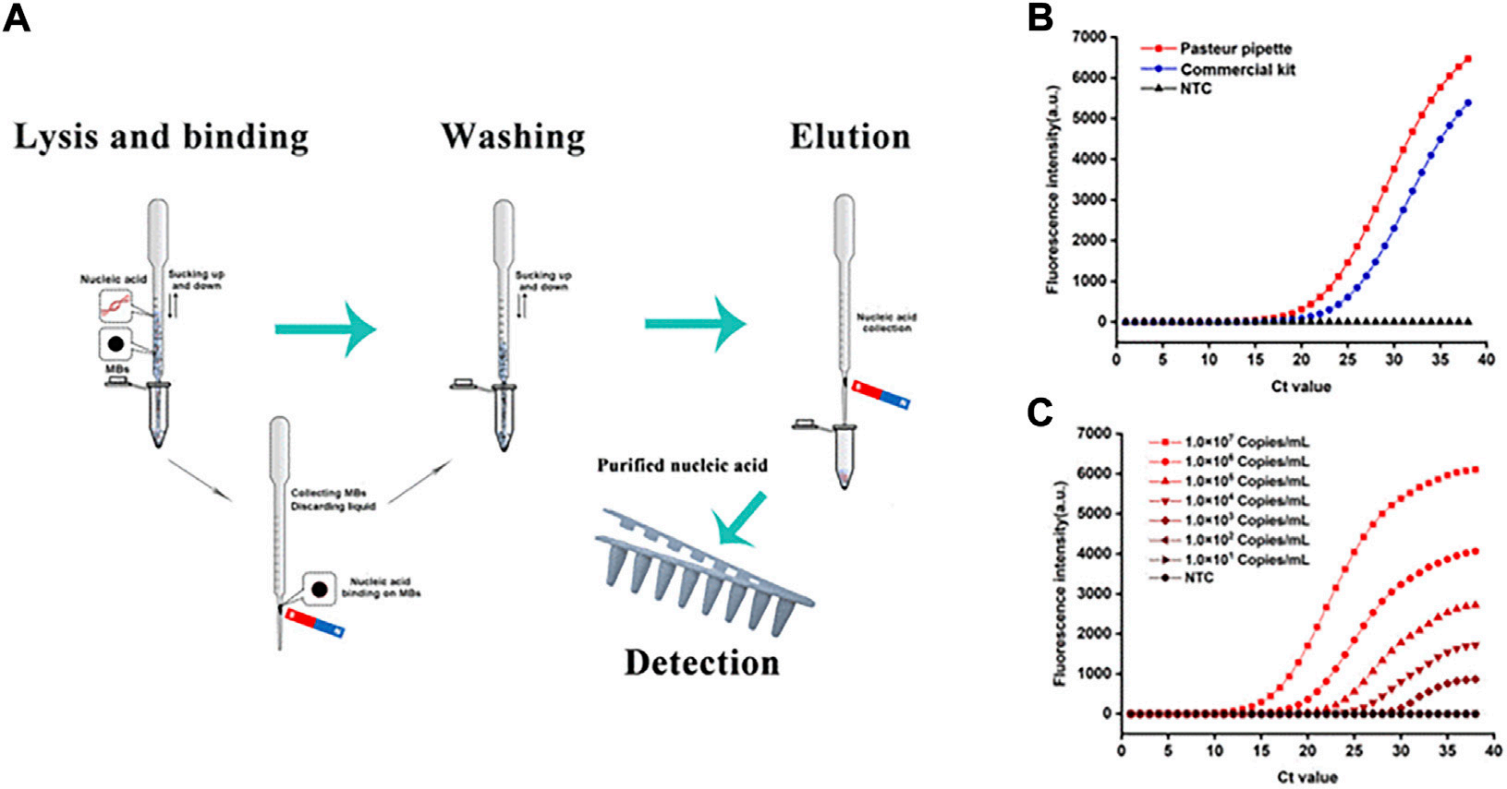
Nucleic acid extraction in Pasteur pipette system and detection results. **(A)** The schematic illustration of lysis and binding step, washing step, elution step and nucleic acid extraction in Pasteur pipette system, and detection step. **(B)** Comparison of extraction effect between Pasteur pipette system and the commercial kit. **(C)** The sensitivity of the Pasteur pipette system. Copyright 2021 Elsevier Inc.

The application of nanomaterials can speed up and automate sample purification, which also helps users simplify sample pre-processing and hence improve accuracy in the POCT procedure. Magnetic nanoparticles have a pivotal position, particularly in NA purification (He et al., 2017; Tang et al., 2018; Dong et al., 2020). A variety of extraction methods involving magnetic nanomaterials are emerging. Kang et al. (2021) developed a simple NA purification system performed in a plastic Pasteur pipette, using magnetic nanoparticles that enter the NA extraction process, as shown in Figure 2. Unlike traditional approaches, the system could complete NA extraction from various samples, including swabs, serum, milk, and pork, in 15 min without assistance from electrical instruments, and the obtained extract had a lower limit for subsequent amplification detection. The system satisfies the requirements for POCT and offers a new idea for integrated detection in POCT devices.

There is a similar study from Pearlman et al. (2020), and work is ongoing to improve the capturing ability of magnetic beads. Bacteria have a different cell surface hydrophobicity, and when the contact angle of the material and water is approximately 90°, the highest bacterial adhesion is shown (Krasowska and Sigler., 2014; Li et al., 2019). In line with this feature, Lee et al., (2021) synthesized tryptamine-functionalized magnetic nanoparticles. Their strategy was to adopt the enrichment of bacteria before DNA extraction. The result demonstrated the extraction efficiency of 1 CFU/ml was 10 times better than that of the commercial kit, and that the process had good stability and accuracy. A novel g-Fe_2_O_3_/PEDOT hybrid nanocomposite was synthesized by da Silva et al. (2021). Here modified nanomagnetic beads were used to extract the DNA from blood and bacteria, and the yields after verification were 6.4 mg and 7.3 mg, respectively, and the captured DNA could be directly used for downstream experiments, such as PCR amplification, without being detached from the nanocomposite.

**FIGURE 3:**
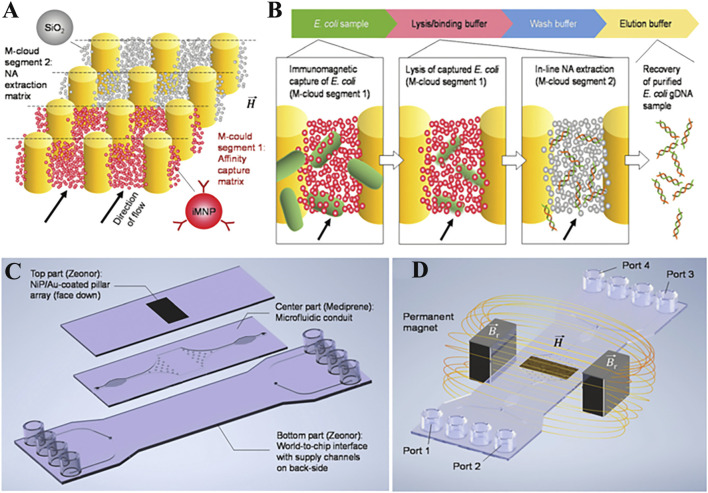
Concept of *in situ* microbial capture and DNA isolation. **(A)** Schematic illustration of segmented, multifunctional M-cloud configuration used within this work. **(B)** Flow chart detailing the order in which assay components are introduced to the M-cloud, along with illustrations of the main steps. **(C)** Schema of the microfluidic chip developed for process integration. **(D)** Illustration of the experimental setup used to generate M-cloud capture regions inside the microfluidic chip. Copyright 2022 Royal Society of Chemistry.

The limitation of magnetic nanoparticles is their slow response to external magnetic stimuli, which means that they need to form larger magnetic complexes by using high nanoparticle concentrations or have long incubation times (Burklund et al., 2020). Poncelet et al. (2021) developed suspended magnetic nanoparticle assemblies (M-clouds) for DNA extraction. The detailed principle is shown in Figure 3. The M-clouds have a strong magnetic field, and can enable almost instant contact between capture sites and the target analyte when the sample solution is flowing. The real result shows that the efficiency of *E.coli* capture and DNA extraction by M-cloud devices was in the range of 17.3–34.1% when the concentration was 10^3^ CFU/ml in environmental water samples. Extraction reagents surely also affect the efficiency of DNA capture (Tang et al., 2013; He et al., 2019). In short, the purpose of pre-test treatment for complex samples is to improve the purity and yield of NA, and all the work has been done to this end. The purification and enrichment of bio samples to obtain high quantity NA is crucial for downstream detection and analysis. Pre-processing of complex samples is therefore essential, and from proper processing it will be easier to obtain highly accurate detection results.

## Detection

Obtaining high-quality DNA is the first step in optimizing detection results, but improvement in the sensitivity and specificity of detection should not be ignored. Therefore, researchers are taking great pains to improve detection methods in order to obtain more exact outcomes. At present, there are two methods for NA detection: amplification and non-amplification. They each have their advantages and disadvantages, which are unavoidable to some extent. Researchers strive to improve these advantages and reduce the disadvantages. The use of nanomaterials can help to address some of the key issues, and in the next section we mainly introduce NA detection methods based on use of nanomaterials in POCT devices.

### Amplification

Exponential amplification in NA detection helps researchers obtain sufficient information from low NA levels. Zhang W et al. (2022) reviewed the application of different nanomaterials (0D, 1D, 2D, and 3D) in isothermal NA amplification, and identified the following advantages of nanomaterials: increasing the activity and stability of the amplification enzyme, activating the activity of enzymes, inhibiting nonspecific amplification, and promoting helicase-dependent denaturation. These advantages are all driving forces in promoting the development of POCT devices.

POCT devices have some limitations, such as complex temperature controls, considerable weight, and high background signals. To solve these problems, more novel technologies have been developed. For example, in the work of Lee et al. (2020), a novel system based on a nanogap-embedded, active whispering-gallery mode resonator was developed to detect the product of recombinase polymerase amplification. Resonators are the core components of detection systems. At present, nano-gap active resonators, disk resonators, and ring resonators are the most common. Ultra-high quality factor optical resonators (Chen et al., 2017) are among the disk resonators, while silicon microring resonators (Park et al., 2013) are ring resonators. Detailed parameter comparisons are given in Table 1, with nanogap active resonators possessing higher sensitivity and lower background signals compared to other resonators. Meanwhile, the author thinks the system can become a POCT device by combining it with a visible LED source, because the size and performance of the entire system fits the demands for POCT devices. Direct detection without NA extraction has become a hot topic, because of the simplified detection steps, shortened detection time, and decreased detection costs, although distractions in the sample or impure NA will affect the detection results.

**TABLE 1:** Comparison of the nano-gap active resonator with other resonators.

Types	Nano-gap active	Disk resonator	Ring resonator
	Resonator		
Pump source	LED/CW laser	Tunable laser	Tunable laser
Coupling scheme	Light illumination in free-space	Tapered fiber	Bus waveguide
Primer state	Immobilized	Immobilized	Immobilized
Amplification condition	Isothermal (38 or 43°C)	Not tested	Isothermal (38 or 43°C)
Background signal	No	Yes	Yes
Target detection time	10 min	20 min	20 min
Detection limit (copies/reaction)	10^0^	10^1^ to 10^2^	10^1^ to 10^2^

**FIGURE 4:**
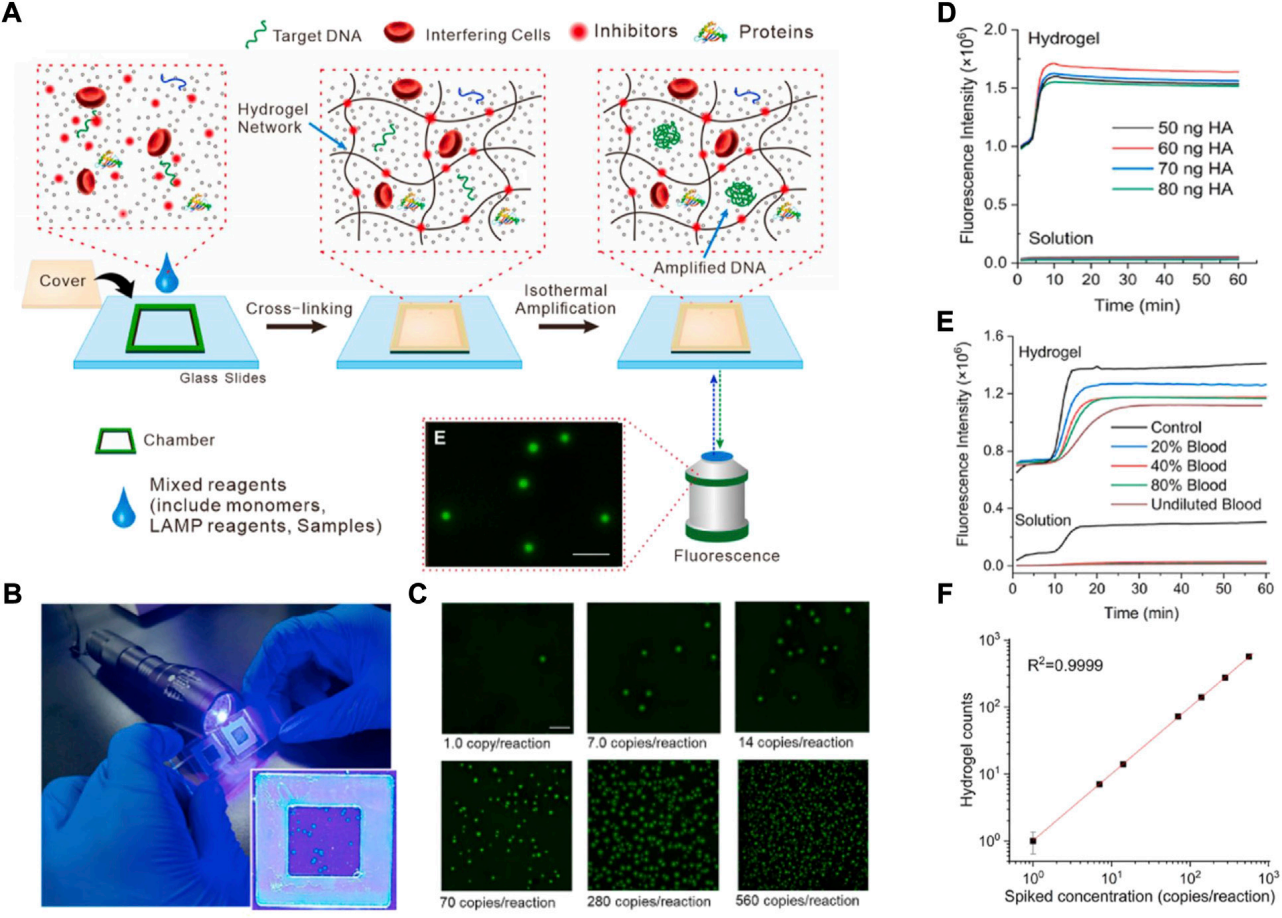
**(A)** Schematic illustration of isothermal amplification inside the nano porous hydrogel for digital LAMP in complex matrices. **(B)** The photograph for naked-eye counts of amplicon dots in the hydrogel under the LED flashlight. **(C)** Endpoint fluorescence images of hydrogel after LAMP reaction for unprocessed whole blood spiked with a series concentration of the *E.coli* genome. Scale bar of 1 mm. **(D)** The impact of different concentrations of whole blood on LAMP reaction performed in the hydrogel or solution. **(E)** The impact of different concentrations of HA on LAMP reaction in the hydrogel or solution. **(F)** Comparison of hydrogel counts to the spiked *E. coli* genome concentration in unprocessed blood. LAMP: loop-mediated isothermal amplification. Copyright 2021 Elsevier B.V.

Similarly, Yi et al. (2021) have synthesized a nanoporous hydrogel with self-cleaning properties for application to direct NA analysis without the need for any sample pretreatment. The detection principle is shown in Figure 4. The inhibitor was adsorbed and removed by the formation of a cross-linking network structure of hydrogel, and next an amplification reaction was produced. The method gives us the new idea that nanoporous hydrogel can become a sort of “dustman” in removing some adverse factors and achieving an efficient NA amplification reaction. Appropriately amplifying the detection signal is necessary. For example, Yang et al. (2020), reviewed detection signal amplification strategies for nanomaterial-based photoelectrochemical (PEC) biosensors, and put forward PEC instruments that are simpler, more cost-effective, with higher analytical detection performance and easier miniaturization. In addition, the PEC sensors can reduce background signals, and have higher sensitivity than conventional electrochemical methods. Alafeef et al. (2021) synthesized the antisense oligonucleotide (ASO)-capped gold nanoparticles (AuNPs) to detect the product of loop-mediated isothermal amplification. By using clinical sample evolution, the accuracy, sensitivity, and specificity of the detection were >98.4%, >96.6%, and 100%, respectively, with a detection limit of 10 copies/μL.

**FIGURE 5:**
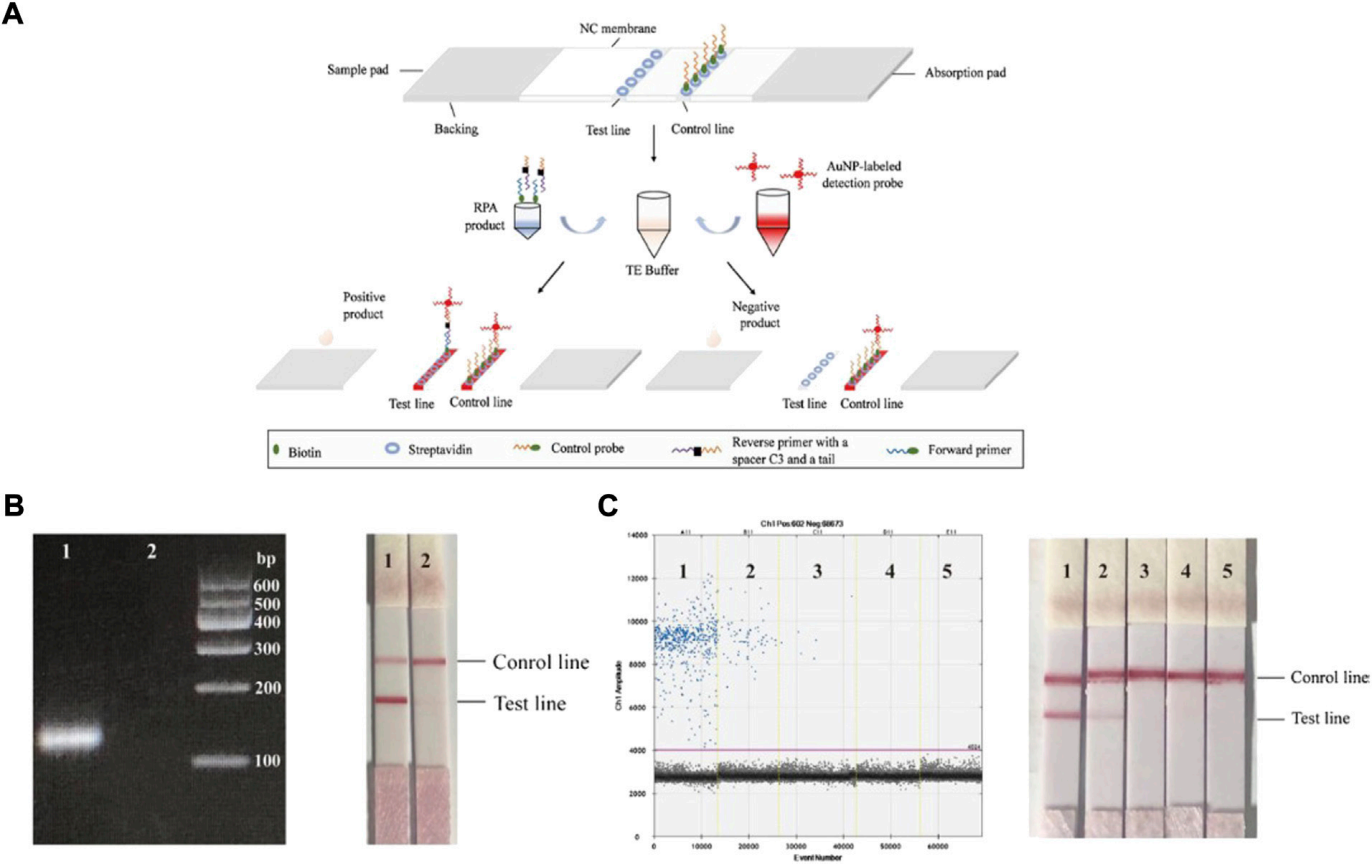
**(A)** The principle of lateral flow gene assay. The RPA product and the AuNP-labeled detection probe were added to TE buffer, and then the mixed droplets were added to the sample pad of the strip. In the presence of positive products, there will be two red lines on both the test line and control line. In the presence of negative products, there will be only one red line on the control line. **(B)** The detection result of sample, Lane 1: target DNA; Lane 2: double-distilled H_2_O. **(C)** A series of tenfold DNA diluents from 10^1^ to 10^4^ extracted from blood infected with ASFV were used to determine the LOD, Lane 1: 10-fold dilution; Lane 2: 10^2^-fold dilution; Lane 3: 10^3^-fold dilution; Lane 4: 10^4^-fold dilution; Lane 5: NTC. Copyright 2022 Springer Nature Switzerland AG.

Rolling circle amplification (RCA) is an isothermal NA amplification method and has ultrahigh specificity for nucleotide variants. Tian et al. (2020), performed two rounds of RCA, and the product of the second round of RCA combined magnetic nanoparticles to generate amplicon coils. The opto magnetic signal can then be charged with the increased amplification product, thus achieving real-time NA detection. Similarly, Wang et al. proposed a lateral flow gene assay (LFGA), combining recombinase polymerase amplification (RPA) and nucleic acid lateral flow (NALF), to directly detect a DNA sample using an oligonucleotide probe. The detection process is described in detail in Figure 5. AuNPs were applied as a dye in RPA amplification product analysis. The whole detection process was completed under 37–42°C in 30 min. This work provides a portable POCT platform without the need for any costly instruments (Wang Z et al., 2021).

These results show that nanomaterials make a contribution to POCT devices in the matter of NA amplification detection, mainly concerning amplified detection signals and reduced nonspecific amplification, and a large body of literature supports this result (Jiang et al., 2013; Nie et al., 2014; Yang et al., 2017; Jiang et al., 2018; Wu et al., 2020a). Overall, nanomaterials have made an outstanding contribution in optimizing NA amplification, and can not only improve the accuracy of detection, but can also shorten detection time and optimize the detection process. Amplification detection requires enzyme assistance, and enzymes need a separate chamber in order to work, and specifically, most reactions require temperature to activate the enzymes. This means that POCT products need to be larger to some extent and are more difficult to operate. However, the above methods can be applied to miniaturized instruments to achieve POCT detection.

### Non-amplification

Direct detection of NA without amplification is more suitable for POCT devices. Meanwhile, the requirement for sample purification is also lower without the involvement of an amplifying enzyme. Nevertheless, here the greatest limitation is low sensitivity, and how to capture more NA in samples and collect as many real signals as possible are some of the problems that researchers need to think about (Mondal et al., 2021; Sun 2022). AuNPs can be designed to suit diversified functionalization options, due to easy surface modification (Lai et al., 2018; Sarfraz and Khan, 2021; Li J et al., 2022b; Liu Y et al., 2022).

**FIGURE 6:**
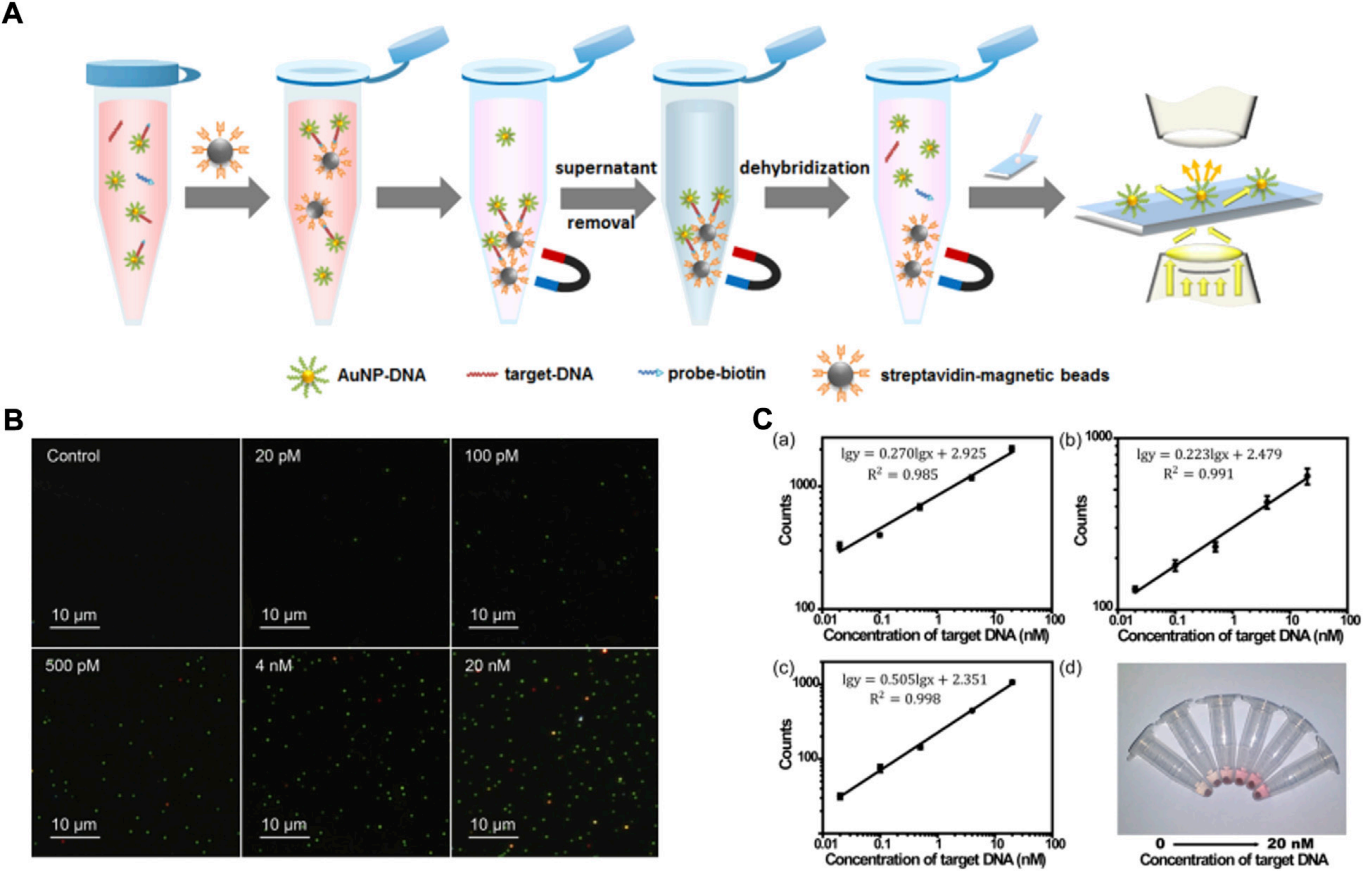
**(A)** The schematic illustration of nucleic acid extraction method combining the sandwich-type DNA hybridization strategy and magnetic separation. **(B)** Dark-field images of AuNP-DNA with different concentrations of 40-nucleotide target DNA. **(C)** Automatic counting results for target DNA with different lengths: (a) 24 nucleotides, (b) 40 nucleotides, and (c) 60 nucleotides; (d) the photograph of the supernatant with different concentrations of 40-nucleotide target DNA (from left to right, 0 p.m., 20 p.m., 100 p.m., 500 p.m., 4 nM, and 20 nM). Copyright 2016 American Chemical Society. Copyright 2021 Elsevier B.V.

AuNPs have the feature of localized surface plasmon resonance, which give them a signal 10- to 10,000-times higher than fluorescent dyes or quantum dots, and this can be detected in simple dark-field microscope. Li et al. (2016) developed a nonamplification sandwich assay for NA detection, and the detection principle is shown in Figure 6. The result shows the length and concentration of target DNA: while the dose of AuNPs can affect the detection efficiency, the detection rate of the target DNA is almost unaffected in a complex matrix containing a variety of proteins and other contaminants. Interestingly, the limit of detection of a clinic sample is 3 fM, and only requires a simple dark-field microscope, which shows that the assay has high simplicity and sensitivity. Ota et al. (2021) used 4-cyano-N-(2-mercaptoethyl) benzamide (4CMB) and oligonucleotides to modify the surface of gold nanorods and finally form a sandwich structure which can detect the target DNA under 1 nM. The limitations of the structure are that the concentration of target DNA is unquantifiable and needs the auxiliary detection of a micro-Raman spectroscope.

A DNA nanostructure is a technique for artificially designing and producing useful NA structures, which, as a bridge, connect the relationship between optoelectronics, thermology dynamics, and genetic information (Zhang et al., 2020; Henry and Stephanopoulos, 2021; Li S et al., 2021; Qin et al., 2022). Although not active, a DNA nanostructure has these advantages: low toxicity, high resistance to degradation in biological media, and an ideal ability to enter cells without transfection agents. These advantages make it a good tool for detecting the NA in live cells (Zhou et al., 2021; Li G et al., 2022a; Li G et al., 2022b). Li et al. (2019) assembled a DNA tetrahedral structure using oligonucleotides to protect the DNAzyme and fluorescent probe. To prevent the uncontrollability of the DNAzyme, the author designed a unique structure to lock its activity, with specific miRNA as the “key” to activating the DNAzyme. The DNAzyme can obtain the dissociated fluorescent signal by separating out the fluorescence-quenched structure, thus achieving the purpose of fluorescence detection. This detection method, compared with its nonamplification molecular beacon counterpart, exhibited at least 10 times higher detection sensitivity (the limit of detection is 16 p.m.) and was able to distinguish miRNA targets from corresponding family members. Of course, the structures can not only detect the miRNA, but are also applied in NA detection, especially short fragment NA. Non-amplification makes NA detection convenient and speedy, and with its shortcomings addressed by some of the above work, is an important part of the development of POCT technology.

In summary, non-amplification NA detection still has huge difficulties, especially for samples with low levels of NA, but it has advantages for direct detection in complex samples. Therefore, it is important to select appropriate detection methods for POCT products, given the method has an important relationship with the samples tested. When developing new POCT products, it is essential to consider the object for detection.

## Data display

In this section, we only discuss the forms of data presentation, and not how the data is processed. The data display has very little to do with the nanomaterial, but it is an integral part of POCT, especially in terms of the user experience. Currently, the personal computer (PC) and built-in touch screen are the two main forms for presenting data visualization, but their disadvantages cannot be ignored. For example, the PC is an expensive and bulky device compared with some POCT products, which is bad news for users. The built-in touch screen solves the problem of needing the supplementary PC, but the use of screens in POCT devices, while on the one hand increasing the instrument’s size, on the other offers a poor user experience.

**FIGURE 7:**
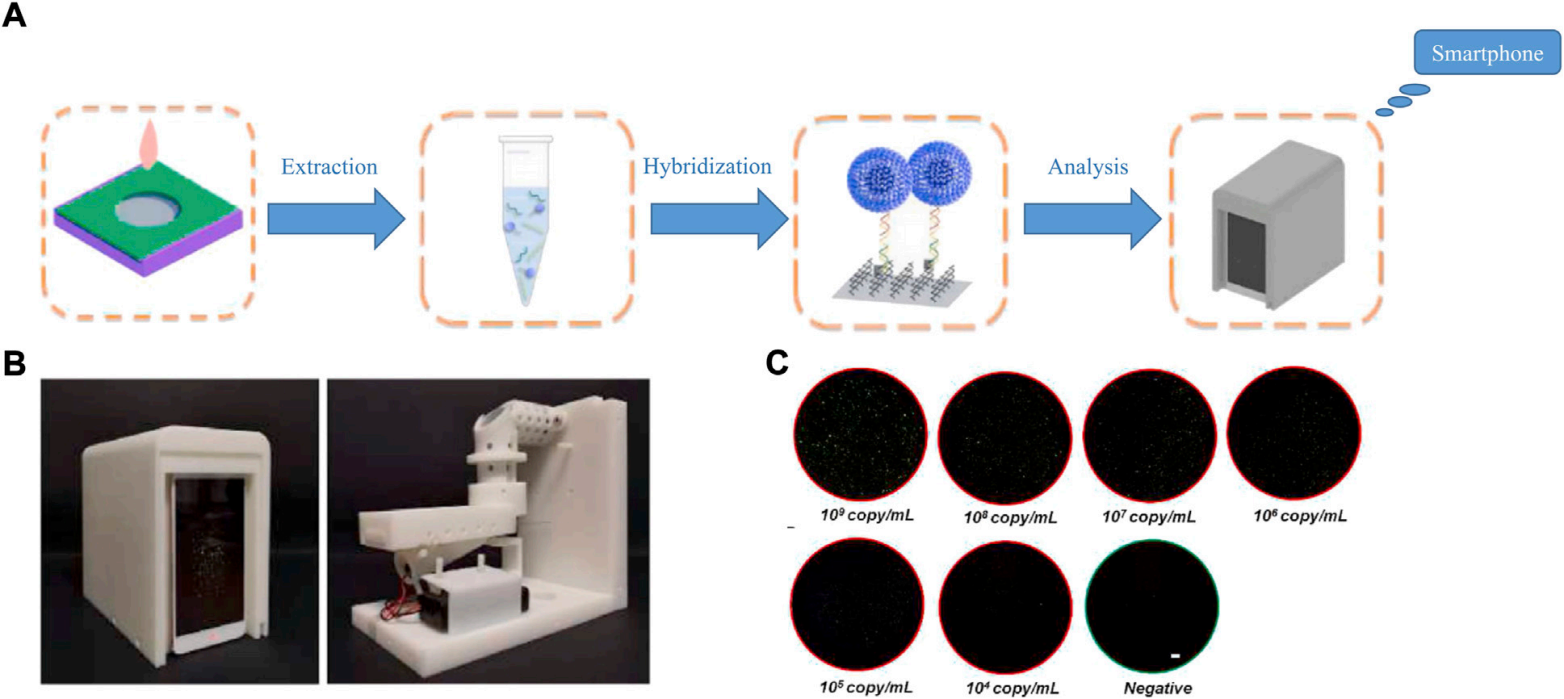
**(A)** Flow chart of the amplification-free smartphone-based detecting technique. **(B)** Portable fluorescence nucleic acid detector. **(C)** Captured FoVs corresponding to different virus concentration. White bar represents 1 μm. Copyright 2021 Elsevier B.V.

Given its popularity, the smartphone is a good choice as a tool for data display. Its characteristics, such as simplicity, affordability and user-friendliness, makes it acceptable to the public. Meanwhile, three data transmission modes, wired, wireless, and Bluetooth, can meet user needs in different situations. Furthermore, smartphones are widely used as detector and analysis devices (Pujari 2021; Pujari et al., 2021; Zhang Z et al., 2022), due to their built-in high-definition cameras. Li Y et al. (2021) built a novel optical detection system for use with a smartphone. Fluorescent images are taken from the camera of a smartphone, and the application is driven by an Android smartphone to analyze the fluorescent signal, and ultimately obtain detection results. The specific detection process and results are shown in Figure 7. Liu S et al. (2022) did the same thing, choosing SYTO9 as a fluorescence reagent to stain the amplification product, which was then observed and analyzed by smartphone. Their result was exciting, and the limit of detection for pathogenic bacteria was 6 copies/mL in the green channel, with the whole process carried out under a constant temperature. Powerful smartphones have brought benefits to productivity, and the lives of people in all aspects, and provide a new idea for the development of POCT. The strong fluorescence of nanomaterials (Li et al., 2018; Mei et al., 2021; Ye et al., 2021) means they can be accurately captured by smartphones, which might create a trend in POCT development in the future.

However, in order to obtain accurate test results, an adequate fluorescence signal and sensitive detection are indispensable. It is also worth noting the smartphone is not professional camera equipment, and can experience data loss or sensitivity decrease, and should thus be treated with caution when used as a detector.

## Discussion and conclusion

Nanomaterials have attracted increased attention due to their unique physical and chemical properties, generating considerable interest in many fields, including biotechnology, catalysis, electronics, optics, and drug-loading (Liu et al., 2020; Gong L et al., 2021; Zhang M et al., 2021; Choi et al., 2022; Gao et al., 2022; Pang et al., 2022). In this review, we focused on the area of NA detection in POCT devices. There is no doubt that the high efficiency and accuracy of NA detection can greatly improve the performance of POCT devices. It is thus important to obtain high quality NA and highly sensitive identification processes. Nanomaterials play a crucial role in NA detection, with detection results showing high performance in terms of sensitivity and selectivity. A lot of the literature reveals that modified nanomaterials not only improve detection capability (about 10–1000 fold compared to routine PCR), but also address the complexity of the sample (Chen et al., 2020; Fang et al., 2021; Liu et al., 2021; Zhang C et al., 2022), such as in the detection of *Salmonella* Typhimurium in complex food environments using a novel fluorescent platform of DNA-stabilized silver nanoclusters. The authors assessed the effectiveness of the main method in terms of sensitivity, specificity, and detection rate in complex samples and their results showed that the detection rate had a linear concentration range of *Salmonella* Typhimurium from 4.6×10^2^ to 4.6 × 10^7^ CFU/ml, with the limit of detection reaching 6.6 × 10^2^ CFU/ml in food samples (Yang et al., 2021). When novel nanomaterials are applied in NA detection, the detection limit for biomolecules is one of the key parameters in evaluating the feasibility of the assay. Hence researchers want to improve the sensitivity of detection by combining the specificity and versatility of NA with the intrinsic properties of nanomaterials. In summary, the design of nanomaterials has become a primary research goal in the biomedical field. With advancements in the artificial intelligence age, new smart technologies have an indispensable place in POCT devices. Sow et al. (2020) summarized the advantages of using smart materials in different work stages of POCT devices, but the challenge presented by high signal-to-noise ratio and accurate detection of analytes in the POCT platform still exists. In general, the main means of promoting the POCT industry in the future will be to use the characteristics of the nanomaterials combined with suitable detection methods.

At the same time, the limitations of the use of nanomaterials also attracts the attention of researchers. Toxicity, stability, and biocompatibility are major problems hindering *in vivo* application, and when it comes to *in vitro* testing, stability is the primary consideration (Ahmed et al., 2021; Jobdeedamrong et al., 2021; Li Y et al., 2021; Zhang Q et al., 2021). No matter how well designed, the deviation in repeated experiment results is too large, which leads to the greatly reduced reliability of such experimental results. Agglomeration of nanoparticles is another main factor that may affect their stability (Magesa et al., 2020; Sun et al., 2021; Diephuis et al., 2022). These properties are nevertheless beneficial in many fields, such as vascular tissue engineering (Rahimnejad et al., 2021). In terms of NA detection, aggregation will affect the efficiency of extraction and detection, and cause the failure of the POCT device. Moreover, the environmental pollution caused by the degradation-resistant nanomaterials should not be ignored, for as the food chain circulates, it accumulates, and eventually enters the body and harms human health. Therefore, easily degradable, non-toxic, and highly stable nanomaterials are the focus for researchers, and the key factors driving the sustainable development of the field.

Application of nanomaterials in POCT devices has become a hot topic for research in recent years, owing in part to the impact of COVID-19, which further stimulated POCT device development. POCT devices represent the future trend in the field of molecular diagnosis, given there is no need for a fixed testing site, as reagents and instruments are portable and can be operated in a timely manner. A problem was posed: i.e., the need to ensure that requirements for speed, quality, and cost all are met in COVID-19 detection (Ding et al., 2020). Beach et al. (2021) thought that POCT could become a key diagnostic tool for health-care providers carrying out COVID-19 rapid-testing, and they identified what needed attention when using POCT devices for detection, such as an established quality assurance framework and the evaluation of clinical performance. While POCT devices have many advantages as a detection tool, they are still a long way from reaching the diagnostic market and being widely used. Therefore, according to the WHO-defined ASSURED criteria, integrating appropriate detection methods to be applied in different fields will shape the direction of our efforts.

Operable paper-based POCT devices have advantages, such as self-driven flow, high surface to volume ratio, and superb biocompatibility. Researchers have thus intensively studied these, and some have produced products that have become commercialized (Liu et al., 2021). For example, Jia et al. (2021) commented on applications of a paper-based POCT device, putting forward reagent storage and sample preparation as the key research directions for the device, in the hope it would have high accuracy while basically meeting WHO’s ASSURED criteria through the endeavors of academia and industry.

Overall, we mainly introduced herein the application of POCT devices to NA testing. The detection aspect is crucial in POCT devices, owing to the development of detection technology closely related to obtaining high quality data. Of course, the other two aspects, loading sampling and data display, cannot be ignored either. Nanomaterials have been widely used in POCT devices, so we have summarized their advantages and use in various parts of these devices, so that researchers might learn about the latest developments in relation to NA detection. However, POCT devices still have their challenges, including low accuracy of detection, the requirement for specialized reagents, and lack of a uniform evaluation system. Of note, in NA detection, fully enclosed detection can reduce the hazards of nucleic acid aerosols, and hence increase the credibility of the result. Some POCT devices associated with NA detection are only a simple combination of a nucleic acid extraction instrument and detector. Strictly speaking, they do not belong to the category of POCT devices. Therefore, developing uniform industry standards is a priority. Second, improving the accuracy and sensitivity of POCT devices will be the future developmental trend, while the use of nanomaterials will be the intrinsic driving force promoting the development of POCT devices. The review we have presented here will be helpful to researchers in promoting POCT products, and will also provide new ideas for creating better healthcare systems for current and future types of disease detection.
